# Modeling the Influence of Local Environmental Factors on Malaria Transmission in Benin and Its Implications for Cohort Study

**DOI:** 10.1371/journal.pone.0028812

**Published:** 2012-01-04

**Authors:** Gilles Cottrell, Bienvenue Kouwaye, Charlotte Pierrat, Agnès le Port, Aziz Bouraïma, Noël Fonton, Mahouton Norbert Hounkonnou, Achille Massougbodji, Vincent Corbel, André Garcia

**Affiliations:** 1 Institut de Recherche pour le Développement (IRD), Mère et Enfant Face aux Infections Tropicales, Cotonou, Benin; 2 Institut de Recherche pour le Développement (IRD), Mère et Enfant Face aux Infections Tropicales, Paris, France; 3 Faculté de Pharmacie, Université Paris Descartes, Paris, France; 4 Institut des Sciences Biomédicales Appliquées (ISBA), Cotonou, Benin; 5 Centre d'Etudes et de Recherche sur le Paludisme Associé à la Grossesse et à l'Enfant (CERPAGE), Contonou, Benin; 6 Université d'Abomey-Calavi, International Chair in Mathematical Physics and Applications (ICMPA-UNESCO Chair), Cotonou, Benin; 7 Institut de Recherche pour le Développement (IRD), Maladies Infectieuses et Vecteurs, Ecologie, Génétique, Evolution et Contrôle (MIVEGEC, UM1-CNRS 5290-IRD 224), Centre de Recherche Entomologique de Cotonou (CREC), Cotonou, Bénin; 8 Université Paris I Panthéon-Sorbonne, Paris, France; 9 Faculté des Sciences de la Santé (FSS), Cotonou, Benin; Johns Hopkins University, United States of America

## Abstract

Malaria remains endemic in tropical areas, especially in Africa. For the evaluation of new tools and to further our understanding of host-parasite interactions, knowing the environmental risk of transmission—even at a very local scale—is essential. The aim of this study was to assess how malaria transmission is influenced and can be predicted by local climatic and environmental factors.

As the entomological part of a cohort study of 650 newborn babies in nine villages in the Tori Bossito district of Southern Benin between June 2007 and February 2010, human landing catches were performed to assess the density of malaria vectors and transmission intensity. Climatic factors as well as household characteristics were recorded throughout the study. Statistical correlations between *Anopheles* density and environmental and climatic factors were tested using a three-level Poisson mixed regression model. The results showed both temporal variations in vector density (related to season and rainfall), and spatial variations at the level of both village and house. These spatial variations could be largely explained by factors associated with the house's immediate surroundings, namely soil type, vegetation index and the proximity of a watercourse. Based on these results, a predictive regression model was developed using a leave-one-out method, to predict the spatiotemporal variability of malaria transmission in the nine villages.

This study points up the importance of local environmental factors in malaria transmission and describes a model to predict the transmission risk of individual children, based on environmental and behavioral characteristics.

## Introduction

Malaria remains endemic in sub-Saharan Africa although dramatic declines in morbidity have been reported in the last five years across a range of settings [1], [2], [3]. This is associated with the distribution of long-lasting insecticide-treated mosquito nets and a switch to first line artemisinin-based combination therapy (ACT). Nonetheless, the disease's burden is still high in Africa where it is a leading cause of mortality, especially in children of under five years of age [4]: new tools—a vaccine, effective drugs, better insecticides—are still needed together with strategies for their use and evaluation. In addition, improving our understanding of host-parasite interactions is a priority.

Accurately assessing the local risk of transmission is fundamental to the development of a malaria control program. In Africa, transmission levels vary enormously and transmission may be either seasonal or perennial [5]. Differences exist not just between different regions but also at the very local level [6], [7], [8]. Key determinants of local transmission intensity [9], [10] include vector profile, ecology and seasonality [11], [12], all of which will affect the efficacy of control operations. The results of recent studies in two different countries (Ghana and Gabon) point up the importance of high-resolution analysis of local variations when designing and monitoring a malaria control operation [12], [13].

Recent findings showed that small-scale differences within an area may have important consequences when it comes to studying individual responses to a risk of infection or to an intervention, e.g. responses to vaccination may be quite different in children who have not been exposed to the antigen to the same extent [14], [15]. Therefore, localized variations ought to be taken into account when considering the risk of infection in a population and the determinants of individual variability (i.e. behavior, physiology and genetics).

This applies to the consequences of placenta-associated malaria (PAM) on the development of specific immunity to *P. falciparum* and the lag before appearance of the first infection in newborns. Four studies showed that children born to mothers with PAM have a higher risk of infection during their first months of life, pointing to the phenomenon of immune tolerance [16], [17], [18], [19]. However, these studies failed to take spatiotemporal variations into account with no entomological or environmental data input into the analyses [20], [21]. Thus, it cannot be ruled out that differences in transmission risk may have affected outcomes, i.e. no conclusion can be drawn about immune tolerance from cohort studies unless information about spatiotemporal variations in malaria transmission is included in the analysis.

This article describes- through the, statistical analysis of the entomological and environmental data of a cohort study conducted in Southern Benin- a new approach to predict the risk of malaria transmission in cohort studies.

## Methods

### Ethics

A written informed consent was obtained from all participants involved in the study. The study protocol was approved by the Ethics Committee of the University of Abomey-Calavi (*Faculté des Sciences de la Santé*; FSS) in Benin and the Consultative Committee of Ethics of Institute of Development Research (IRD).

### Study area

The study was conducted in the district of Tori-Bossito (Republic of Benin), between July 2007 and July 2009. Tori Bossito is on the coastal plain of Southern Benin, 40 kilometers north-east of Cotonou. This area has a subtropical climate and during the study the rainy season lasted from May to October. Average monthly temperatures varied between 27°C and 31°C. The original equatorial forest has been cleared and the vegetation is characterized by bushes with sparse trees, a few oil palm plantations and farms. The study area contained nine villages (Avamé centre, Gbédjougo, Houngo, Anavié, Dohinoko, Gbétaga, Tori Cada Centre, Zébè and Zoungoudo). Tori Bossito was recently classified as mesoendemic with a clinical malaria incidence of about 1.5 episodes per child per year [22]. Pyrethroid-resistant malaria vectors are present [8].

### Mosquito collection and identification

Entomological surveys based on human landing catches (HLC) were performed in the nine villages every six weeks for two years (July 2007 to July 2009). Mosquitoes were collected at four catch houses in each village over three successive nights (four indoors and four outdoors, i.e. a total of 216 nights every six weeks in the nine villages). Five catch sites had to be changed in the course of the study (2 in Gbedjougo, 1 in Avamè, 1 in Cada, 1 in Dohinoko) and a total of 19 data collections were performed in the field between July 2007 and July 2009. In total, data from 41 catch sites are available.

Each collector caught all mosquitoes landing on the lower legs and feet between 10 pm and 6 am. All mosquitoes were held in bags labeled with the time of collection. The following morning, mosquitoes were identified on the basis of morphological criteria [23], [24]. All *An. gambiae* complex and *An. funestus* mosquitoes were stored in individual tubes with silica gel and preserved at −20°C. *P. falciparum* infection rates were then determined on the head and thorax of individual anopheline specimens by CSP-ELISA [25].

### Environmental and behavioral data

Rainfall was recorded twice a day with a pluviometer in each village. In and around each catch site, the following information was systematically collected: (1) type of soil (dry lateritic or humid hydromorphic)—assessed using a soil map of the area (map IGN Bénin at 1/200 000^e^, sheets NB-31-XIV and NB-31-XV, 1968) that was georeferenced and input into a GIS; (2) presence of areas where building constructions are ongoing with tools or holes representing potential breeding habitats for anopheles; (3) presence of abandoned objects (or ustensils) susceptible to be used as oviposition sites for female mosquitoes; (4) a watercourse nearby; (5) number of windows and doors; (6) type of roof (straw or metal); (7) number of inhabitants; (8) ownership of a bed-net or (9) insect repellent; and (10) normalized difference vegetation index (NDVI) which was estimated for 100 meters around the catch site with a SPOT 5 High Resolution (10 m colors) satellite image (Image Spot5, CNES, 2003, distribution SpotImage S.A) with assessment of the chlorophyll density of each pixel of the image.

Due to logistical problems, rainfall measurements are only available after the second entomological survey. Consequently, we excluded the first and second surveys (performed in July and August 2007 respectively) from the statistical analyses. However, the results of all 19 entomological catches were included in the descriptive part of the results.

### Statistical analysis

The statistical analysis was conducted in two phases.

First an explanatory regression model was constructed to determine the correlation between *Anopheles* density and the above-mentioned environmental factors. On the basis of these results, a predictive model was constructed to predict spatiotemporal malaria transmission in houses for which environmental but not entomological data were available. The error distribution of this model was compared with that of a simple “pragmatic” model based on real entomological data.

#### Variables

The dependent variable was the number of Anopheles collected in a house over the three nights of each catch, and the explanatory variables were the environmental factors, i.e. the mean rainfall between two catches (classified according to quartile), the number of rainy days in the ten days before the catch (3 classes [0–1], [2–4], >4 days), the season during which the catch was carried out (4 classes: end of the dry season—February to April; beginning of the rainy season—May to July; end of the rainy season—August to October; beginning of the dry season—November to January), the type of soil 100 meters around the house (dry or humid), the presence of constructions within 100 meters of the house (yes/no), the presence of abandoned tools within 100 meters of the house (yes/no), the presence of a watercourse within 500 meters of the house (yes/no), NDVI 100 meters around the house (classified according to quartile), the type of roof (straw or sheet metal), the number of windows (classified according to quartile), the ownership of bed nets (yes/no), the use of insect repellent (yes/no) and the number of inhabitants in the house (classified according to quartile).

#### Explanatory model

Since the dependent variable was a count, in order to take into account the hierarchical structure of the data (repeated catches in the same house, four sites per village) with correlation possible between the entomological measurements, a Poisson mixed model was constructed with three random intercepts at the village, site and catch levels, i.e. for the k^th^ catch in the j^th^ site in the i^th^ village:
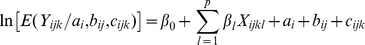
where Y is the number of collected *Anopheles*, X is a p-vector of environmental variables, β is a (p+1)-vector of the model's parameters (including the fixed intercept β_0_), a_i_ is the random intercept at the village level, b_ij_ is the random intercept at the site level and c_ijk_ is the random intercept at the catch level. It can be shown that in this model
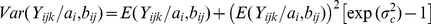
Then 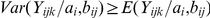, showing that adding the random intercept at the catch level is a way to preclude residual over-dispersion of the model with only two random intercepts at the village and site levels.

All environmental variables were first introduced in the model, and a backward procedure was applied to select only those that remained significant in the final model.

To achieve the most parsimonious model, adjacent classes of a categorical variable were grouped together if the corresponding regression estimates were close.

#### Predictive model

A regression model was then constructed to predict *Anopheles* count when only environmental data are available.

The model was selected using a leave-one-out method (e.g. see [26]). For a given set of covariates X, the following steps were repeated for all catch sites j from 1 to 41:

- A regression model of the number of *Anopheles* collected versus environmental variables was performed using the observations from all sites except the i^th^ (i.e. by excluding the 17 data collections of the i^th^ site)
- This model was used to predict a number of *Anopheles* collected P_jk_ (k in 1… 17) in the 17 data collections at the i^th^ site using the corresponding known environmental covariates for the i^th^ site and the coefficients of the model from the above step
- The prediction errors E_jk_ = |Y_jk_−P_jk_|/(Y_jk_+1) were computed

Once the algorithm had been applied for the 41 sites, the median of the 612 prediction errors (all catches at all sites) was determined, and the final set of covariates was the one with the lowest median prediction error.

After the selection of variables, interaction terms were introduced and conserved in the final model if they led to a lower median prediction error.

In this prediction model, correlation between the observations was taken into account by entering a “village” variable in the model. The general equation of this model was then:
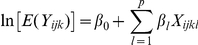
where Y is the number of collected *Anopheles*, and X is a vector of environmental variables (including the “village” variable).

In order to evaluate our model's ability to estimate the spatiotemporal pattern of malaria transmission, we compared the model's predictions to the observed number of *Anopheles* collected in the field.

We also used another predictive “pragmatic” model in which the predicted number of collected *Anopheles* for the k^th^ catch in the j^th^ site in the i^th^ village was estimated by the mean number of *Anopheles* collected at the other three sites in the same village during the same catch, e.g. during the first survey in Gbetaga, 4, 7, 7 and 26 *Anopheles* were caught in the four catch sites respectively. The numbers of collected *Anopheles* predicted by this pragmatic model were then (7+7+26)/3, (4+7+26)/3, (4+7+26)/3 and (4+7+7)/3 in the four houses respectively. Then, we compared the distribution errors obtained from the two models (predictive and pragmatic) according to the predicted *Anopheles* count.

All statistical analyses were performed by using STATA software version 10 (Stata Corporation, College Station, Texas, United States).

## Results

**Figure 1:**
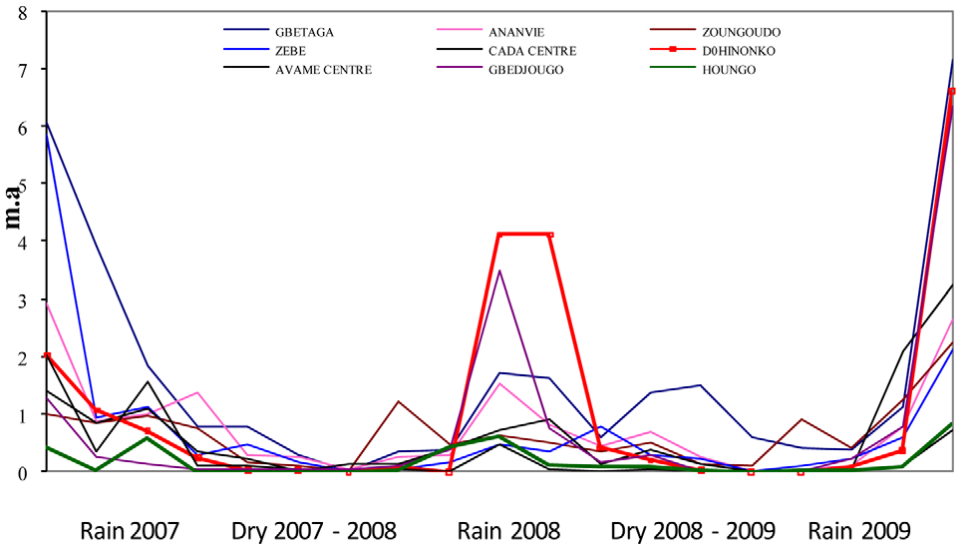
Number of *Anopheles gambiae* s.l. collected per man per day (ma) in the 9 villages for each of the 19 surveys.

**Figure 2:**
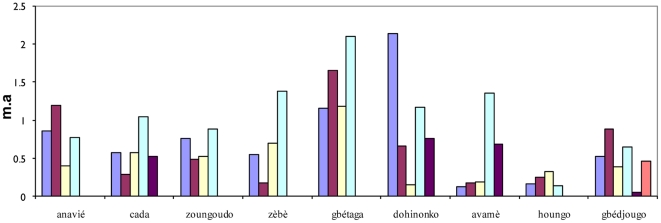
Mean m.a. in the 9 villages. Each bar represents the mean m.a. throughout study in one house.

During the 19 surveys between July 2007 and July 2009, a total of 3,074 malaria vectors were caught (93.3% *An. gambiae s.l.* and 6.7% *An. funestus*). The median number of vectors caught in the 684 collections (19 catches at 4 catch sites in 9 villages), was 1 (interquartile range [0–4], max = 87). Evolution in vector density as defined by the mean number of bites per human per night (m.a) according to time is shown in Figure 1. These findings point to time- and space-dependent fluctuations in vector density. Variation in m.a. was dependent on season and positively associated with rainfall. Spatial differences in m.a. were observed between the 9 villages, particularly during each rainy season (from June to November) even at a village scale, e.g. there was a strong difference in m.a. changes between the 2 villages of Houngo and Dohinoko which are only two kilometers apart (highlighted curves), the first showing a low vector density throughout the study and the second one showing strong seasonal variation with a substantial increase during the rainy season. Furthermore, in all villages (except Houngo) we observed marked m.a. differences between catch sites, reflecting spatial variations in vector density at the site level (Figure 2).

As mentioned above, statistical analyses were conducted for just 17 surveys in which a total of 2,292 malaria vectors were collected.

**Figure 3:**
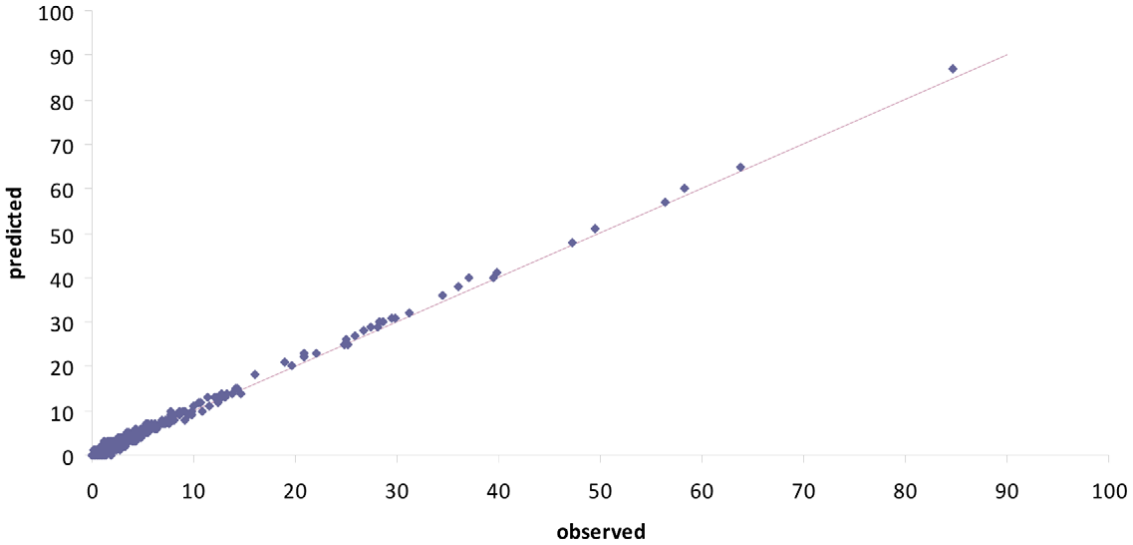
Relationship between observed and predicted numbers of *Anopheles gambiae* collected (explanatory model). The straight line is the bisector.

Table 1 shows the final multivariate explanatory model. This model contained random intercepts at the village, site and catch levels, each random intercept improving the likelihood of the data. Both the mean rainfall between 2 surveys and the number of rainy days in the 10 days before the survey correlated positively with *Anopheles* density, as expected. Independently, season also correlated with *Anopheles* density, with density higher during rains. Several site characteristics correlated with higher vector density: proximity to a watercourse, a dry soil, and a higher NDVI (vegetation index). All these results point to local spatiotemporal variations in malaria transmission. Figure 3 shows a very good adjustment between the number of *Anopheles* collected at each survey and the explanatory model's predictions.

**Table 1:** Environmental factors associated with the density of malaria vectors at Tori Bossito, Benin (explanatory Poisson mixed model).

Fixed effects	Estimation	Standard error	p-value
Watercourse No	.	.	
Yes	1.86	0.63	0.003
Type of soil Humid	.		
Dry	2.27	0.72	0.002
NDVI Low	.		
High	0.46	0.23	0.05
Season End of dry season	.	.	<10−3
Beginning of rainy season	1.63	0.18	
End of rainy season	0.44	0.17	
Beginning of dry season	−0.49	0.19	
Mean rainfall Low	.	.	<10−3
High	0.99	0.23	
Number of raining days before collection(1)			
[0–1]	.	.	<10−3
[2–4]	0.34	0.17	
>4	0.70	0.20	
**Random intercepts (standard error)**			
Village level	0.71	0.19	
House level	0.21	0.11	
collection site level	1.04	0.06	

(1) 10 days period before the mosquito collection.

Therefore, when entomological data are not available, a predictive model based on environmental data could be useful to estimate the spatiotemporal entomological risk in a house.

**Figure 4:**
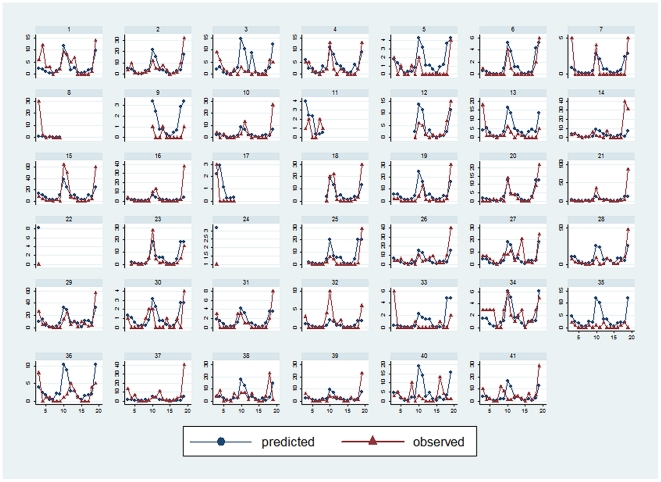
(Predictive model) and observed numbers of *Anopheles* in the 41 houses. Each graph shows the observed (solid line) and the predicted (dashed line) number of *Anopheles* during each catch in a house.

**Figure 5:**
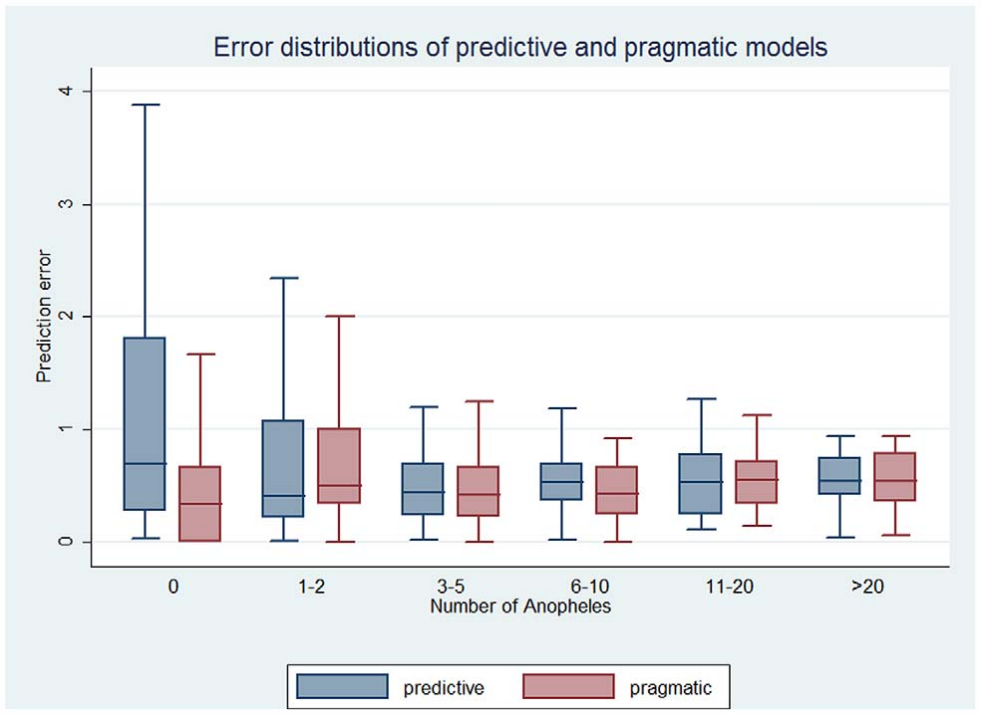
Error distributions of the pragmatic and predictive models according to the number of observed *Anopheles*. In each group (number of *Anopheles*), the left box corresponds to the predictive regression model and the right box to the pragmatic regression model.

The best predictive model contained the following covariates: season, mean rainfall between 2 surveys, number of rainy days in the 10 days before the survey, use of insect repellent, NDVI, and an interaction term between season and NDVI. Figure 4 shows comparisons between the predictions generated with the regression model and the real number of *Anopheles* collected at each of the 41 sites. The model fits with the actual spatiotemporal transmission pattern for most but not all sites. Figure 5 shows a comparison between the error distributions of the regression and the pragmatic models, according to the real number of *Anopheles* collected. The error distributions and hence the predictive powers of both models are highly comparable.

**Figure 6:**
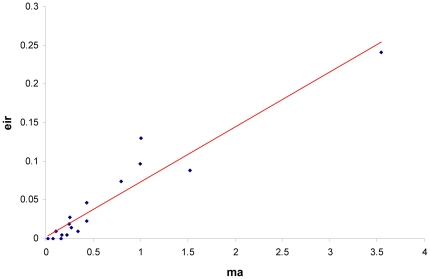
Relationship between mean Entomological Inoculation Rates (EIR) and mean m.a. Coordinates are: x, the mean m.a. of all houses during a catch and y, the mean EIR for all houses during a catch.

The number of infected *Anopheles* was low throughout the study: the average Entomological Inoculation Rate (EIR) was 0.046 infected bite/human/night. When EIR was used as dependent variable instead of m.a., the model failed to converge when too many covariates were introduced together. However EIR and m.a. were highly correlated (see figure 6, r = 0.95). Moreover, when EIR was used as dependent variable with climatic variables—mean rainfall between two collections, number of rainy days during the 10 days before collection, and season—as the only independent covariates, the same pattern was obtained (data not shown). For these reasons we used the total number of *Anopheles* caught on humans (m.a.) for the statistical analyses.

## Discussion

This study set out to investigate the relationship between the distribution of malaria vectors in southern Benin and local environmental and climatic factors (at the level of both village and house), and to propose a predictive model for the spatiotemporal risk of exposure to *Anopheles* mosquitoes.

We observed substantial variations in malaria vector density at the level of both village and house, even between houses which are close together (separated by just a few dozen meters). We found that this variability could be explained not only by conventional climatic factors (rainfall, season) but also certain environmental factors, i.e. a watercourse nearby, and vegetation index and soil type in the immediate surroundings (see also [27]).

The density of malaria vectors and the intensity of transmission are relatively low in this area, confirming previous findings [8], [22]. We showed that EIR and m.a. correlate strongly and, when EIR is used as the dependent variable in our models, the pattern of the results is the same. However, problems of stability and convergence were observed with EIR so m.a. was used for the statistical modeling. Nevertheless, there is no reason why an infected mosquito's behavior—which depends on ecological and environmental conditions—would be any different [28], [29]. Based on this finding, we believe that our model based on *Anopheles* density can accurately predict malaria transmission.

For statistical analyses, some continuous variables (e.g. NVDI, rainfall levels…) were recoded as categorical variables, leading to a loss of information. This loss of information is reduced by using more classes and, furthermore, this method presents a double advantage: the results are easy to interpret between the different classes; and there is no need to assume a linear relationship between the dependent variable and the covariate. Three random intercepts were introduced in our explanatory model, at the village, site, and catch levels. Each random intercept increased significantly the likelihood of the data (with the highest increase afforded at the catch level). This is consistent with the fact that they correctly take into account the hierarchical structure of the data, as well as unobserved variables which could explain the vector's variability at each of the three levels. The quality of the model's adjustment to the data was spectacularly improved by the random intercept at the catch level. This makes it possible to introduce this intercept to deal with over-dispersed data—as recommended by Rabe-Heskett and al. [30]—and confirms the reliability of the model.

The association between vector density and environmental or climatic factors has been widely studied [12], [13], [31], [32], [33], [34] with rainfall and season consistently identified as significant factors. We have identified the number of rainy days before mosquito collection as an additional factor: this could be explained by an increase in the number of temporary water habitats (puddles) favorable to the development of the mosquito larvae. There was no correlation between m.a. and the presence of a net or repellent use in the house, even when the indoor biting rate was considered. This could be explained by the fact that we used a man-landing capture technique without a bed net in a limited number of houses. We also showed substantial variations in vector density, not only between different villages but also in the same village, between houses within a few dozen meters of one another. Factors that could explain this include factors in the houses' immediate surroundings: the presence of a watercourse nearby, a higher vegetation index, and dry soil were all associated with higher vector density. The positive correlation with dry rather than hydromorphic soil could be explained by the fact that the latter is concentrated around the main river in the area, and is overrun by dense aquatic vegetation which prevents *Anopheles gambiae* breeding.

Other studies that have investigated the relationship between domestic features and malaria transmission [35], [36] have shown that roof and ceiling type can also affect malaria transmission. All these observations point up the importance of taking local characteristics—of the village, the house and the house's immediate surroundings—into account when dealing with the variability of malaria transmission.

These findings may also have important consequences when focusing on mechanisms to explain differences in *P. falciparum* infection or the incidence of malaria attacks between groups of individuals when both “environmental” and “biological” determinants are involved. This is particularly relevant for children born to a mother with PAM who tend to develop malaria sooner [16], [17], [18], [19]. The hypothesis of immune tolerance has been put forward to explain this, i.e. fetal exposure to malaria modulates neonatal immunity in endemic areas where infection during pregnancy is common [37], [38]. However, even though immune tolerance is due to infection status of the mother during gestation, its consequences in term of newborn's susceptibility to infection manifests itself through the fact that offspring of mothers with placental malaria at delivery experience their first *P. falciparum* parasitemia at a younger age. However, it also seems self-evident that babies more strongly exposed to *Plasmodium* would be at greater risk of contracting malaria rapidly after birth. In consequence, to demonstrate immune tolerance and evaluate its role in determining susceptibility to early malaria infection, local transmission variations have to be taken into account. This parameter can be addressed by a statistical method that takes into account both spatial and temporal variability but, in the studies cited above, the only exposure-related variable introduced into the Cox model was “area of residence”. Such a variable—which is time-independent and has the same value for all children living in the same village—provides little information about the differential exposure of children in a cohort. After initial demonstration of such local spatiotemporal variability, a novel approach was formulated to predict malaria transmission in houses within a limited area with known ecological and environmental characteristics. We have demonstrated that a regression model including spatial and time-dependent variables at the village and the house levels, yields a spatiotemporal prediction of malaria transmission comparable to that obtained on the basis of entomological data.

This approach constitutes a substantial improvement and the model will be applied to all children in the cohort over the relevant period. The predictions will be used as a time-dependent covariate in analysis of the interval before the first malaria infection (Cox model) to investigate the role of immune tolerance in this parameter.

Finally, this approach can be used to estimate spatiotemporal variations in malaria transmission in cohort studies, thereby delineating and elucidating the respective roles of environment, behavior and physiology in determining susceptibility to infection.
